# PREVIOUS FALLS AND FEAR OF FALLING ON FUNCTIONAL LIMITATIONS: A LONGITUDINAL STUDY

**DOI:** 10.1093/geroni/igac059.146

**Published:** 2022-12-20

**Authors:** Kehan Liu, Wenting Peng, Christina Miyawaki, Chunxiao Li, Yu Zheng, Siyuan Tang, Minhui Liu

**Affiliations:** Central South University, Changsha, Hunan, China (People's Republic); Central South University, Changsha, Hunan, China (People's Republic); University of Houston, Houston, Texas, United States; Central South University, Changsha, Hunan, China (People's Republic); Central South University, Changsha, Hunan, China (People's Republic); Central South University, Changsha, Hunan, China (People's Republic); Central South University, Changsha, Hunan, China (People's Republic)

## Abstract

Previous falls and fear of falling (FoF) are risk factors that affect older adults’ daily activities. However, it remains unclear about their combined effects on functional limitations. Using Round 1 (R1) and Round 2 (R2) data from the National Health and Aging Trends Study, we examined whether falls and FoF in R1 independently and jointly predict functional limitations in R2 and the moderating role of demographic factors in this relationship among community-dwelling older adults aged 65 years and older. Previous falls and FoF were ascertained by asking participants whether they had fallen down in the last year and worried about falling in the last month. Functional limitations included any difficulties with mobility, self-care, or household activities. Poisson regression models were used to analyze data. Of 5,956 participants, 16.4% had falls only, 14.3% had FoF only, 14.5% had both, and 54.8% had neither. In the full adjusted model, those who experienced concurrent falls and FoF had a higher risk of functional limitations than those without falls and FoF (Mobility: Incidence risk ratio, IRR=1.44, 95% CI: 1.33-1.57; Self-care: IRR=1.29, 95% CI: 1.20-1.38; Household tasks: IRR=1.32, 95% CI: 1.21-1.44), as well as those with falls only (Mobility: IRR=1.32, 95% CI: 1.21-1.44; Self-care: IRR=1.26, 95% CI: 1.17-1.35; Household tasks: IRR=1.18, 95% CI: 1.08-1.29) and FoF only (Mobility: IRR=1.38, 95% CI: 1.27-1.51; Self-care: IRR=1.26, 95% CI: 1.17-1.35; Household tasks: IRR=1.31, 95% CI: 1.20-1.43). The findings suggest that strategies to improve falls and FoF together could potentially help prevent functional limitations.

